# Anticancer properties of red beetroot hydro-alcoholic extract and its main constituent; betanin on colorectal cancer cell lines

**DOI:** 10.1186/s12906-023-04077-7

**Published:** 2023-07-18

**Authors:** Amir Saber, Nasim Abedimanesh, Mohammad-Hossein Somi, Ahmad Yari Khosroushahi, Shima Moradi

**Affiliations:** 1 Department of Nutritional Sciences, School of Nutritional Sciences and Food Technologies, Kermanshah University of Medical Sciences, Isar Sq., Across From Farabi Hospital, P.O. Box 6719851552, Kermanshah, Iran; 2 Department of Nutrition, School of Medicine, Zanjan University of Medical Sciences, Zanjan, Iran; 3 Liver and Gastrointestinal Diseases Research Center, Tabriz University of Medical Sciences, Tabriz, Iran; 4 Department of Medical Nanotechnology, School of Advanced Medical Sciences, Drug Applied Research Center, Tabriz University of Medical Sciences, Tabriz, Iran; 5 Student Research Committee, School of Nutritional Sciences and Food Technologies, Kermanshah University of Medical Sciences, Kermanshah, Iran

**Keywords:** Beetroot, Betanin, Colorectal cancer, Apoptosis, Gene expression

## Abstract

Colorectal cancer (CRC) is the third most common type of cancer worldwide. Red beetroot (*Beta vulgaris*) contains Betanin as its major betacyanin, possessing wide proapoptotic effects. This study aimed to investigate the anticancer and pro-papoptotic effects of beetroot hydro-alcoholic extract (BHE) and betanin, on colorectal cancer cell lines. BHE and betanin were used to treat Caco-2 and HT-29 colorectal cancer cells. MTT assay, DAPI staining, and FACS-flow cytometry tests were used to determine the half-maximal inhibitory concentration (IC50) and apoptosis-inducing evaluations. Intended genes were assessed by real-time polymerase chain reaction (RT-PCR). The IC50 for HT-29 and Caco-2 cell lines were 92 μg/mL, 107 μg/mL for BHE, and 64 μg/mL, 90 μg/mL for betanin at 48 h, respectively. BHE and betanin significantly inhibited the growth of both cancer cell lines time and dose-dependently. DAPI staining and flow cytometry results revealed significant apoptosis symptoms in treated cancerous cell lines. The expression level of proapoptotic genes (BAD, Caspase-3, Caspase-8, Caspase-9, and Fas-R) in treated HT-29 and Caco-2 cells was higher than in untreated and normal cells. In contrast, the anti-apoptotic gene (Bcl-2) was significantly downregulated. BHE and betanin effectively inhibited cancer cell proliferation and induced apoptosis via the modification of effective genes.

## Introduction

Colorectal cancer (CRC) is the third most common type of cancer, with approximately 1.2 million new cases, and its incidence and mortality rate have increased among young adults in recent years [1, 2]. In addition to age and male sex, other inherent and environmental factors such as inflammatory bowel disease, smoking, excessive alcohol consumption, high consumption of red and processed meat, obesity, and diabetes have been identified as risk factors [2, 3]. Diet and lifestyle are major contributors to CRC development, and healthy habits such as consuming fruits and vegetables, engaging in regular physical activity, and maintaining a healthy body weight can help prevent 30–35% of cancer cases [4, 5]. Fruits and vegetables are rich sources of bioactive compounds with high antioxidant capacity, such as ascorbic acid, R-tocopherol, carotenoids, anthocyanins, and betalains [6, 7] which can scavenge free radicals that cause cellular damage [8, 9].

**Fig. 1:**
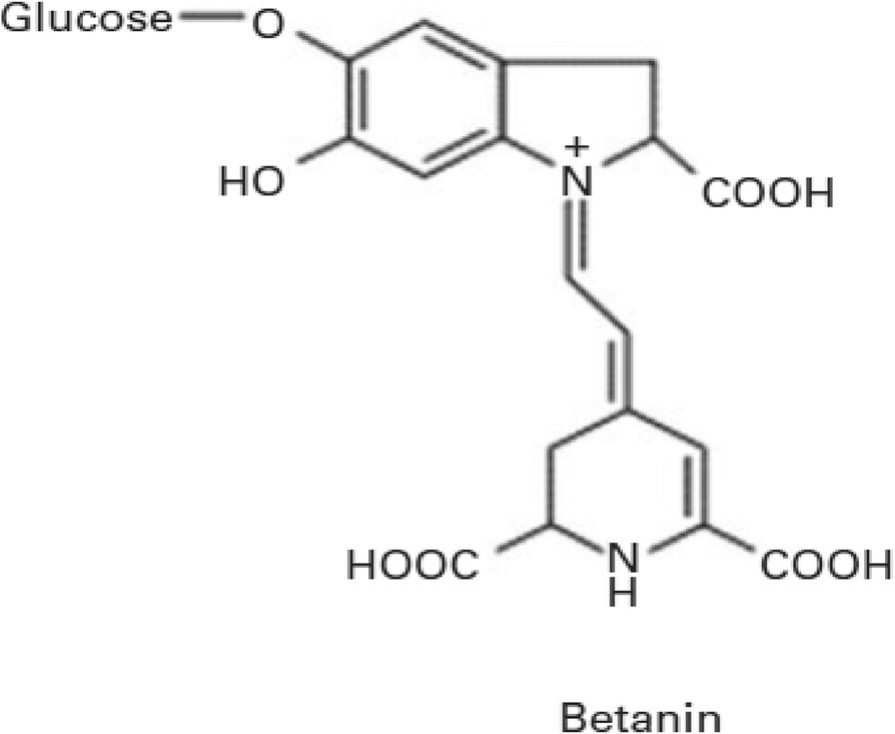
General chemical structure of Betanin (betanidin-5-O-b-glycoside) [13]

Beetroot (*Beta vulgaris* L.) having a place to the Chenopodiaceae family, is high in dietary fibers, minerals (manganese, magnesium, potassium, sodium, phosphorus, iron, zinc, copper, boron, silica and selenium), B complex-vitamins (B1, B2, B3, B5, B6, folate, B12) and also have 50% of phenolic compounds, including betalains, carotenoids, phenols as well as complex carbohydrates, and inorganic nitrate [10, 11]. Also, this vegetable has effective antioxidant activity via different bioactive compounds such as triterpenes, sesquiterpenoids, carotenoids, coumarins, flavonoids (tiliroside, astragalin, rhamnocitrin, rhamnetin, kaempferol), phenolic compound, glycine betaines, beta-cyanlins, saponins, and betalains [10, 12]. Betalains are water-soluble nitrogenous pigments that exist in most plants of the order Caryophyllales, and red beetroot is the rich source of this pigments [13] due to the presence of betalains composed of red pigments (betacyanins) and yellow pigments (betaxanthins) [6, 14]. Numerous studies demonstrated that carotenoids in beetroot like lycopene, β-carotene, and lutein have significant anticancer and chemotherapeutic activity against different cancers [15–19]. Betanin is the main constituent of red beetroot pigments and possesses various favorable biological effects, including antioxidant, anti-inflammatory, hepatoprotective, and antitumor activities [20, 21]. This water-soluble nitrogenous compound comprises 75–95% of red beetroot pigments (300–600 mg/kg) (Fig. 1) [13, 14]. Numerous investigations have shown that beetroot and betanin, can be considered as strong chemopreventive agents that induce apoptosis and decrease cell proliferation, angiogenesis, and inflammation in skin, liver, lung, and esophageal cancers in experimental animals and cancer cell lines [6, 7, 22, 23]. In addition, some other chemopreventive activities of betanin in the form of continuous delay in tumor onset, increase in tumor latency, decrease in tumor multiplicity, and a further reduction in splenomegaly have also been reported [24, 25]. Besides, it was shown that treatment of human chronic myeloid leukemia cell line-K562 with 40 mM betanin decreases cell proliferation (50%) and induces intrinsic apoptosis pathway by activation of caspase-3 as an executioner caspase in apoptotic cascades [26, 27]. In the Traditional Persian Medicine and other medicinal systems including the Arab, traditional Chinese and Ayurvedic medicine beetroot recommended for the prevention and management of metastatic progress of cancer [28, 29]. Besides, in Trinidad and Tobago, beetroot uses as a herbal remedy and functional food by breast, prostate, and colorectal cancer patients [30]. Also, in some cultures and countries, beetroot is being consumed by the majority of gastrointestinal cancer patients to ameliorate chemotherapy treatment side effects and as an alternative dietetic measure [30–32]. In vitro investigations have shown that beetroot extract can have cytotoxic effects on androgen-independent human prostate cancer cells (PC-3) and estrogen receptor-positive human breast cancer cells (MCF-7). Moreover, a synergistic effect was observed when cancer cells were simultaneously treated with beetroot extract and the chemotherapy drug doxorubicin [23, 33]. Different studies showed that Betanin as the main constituent of red beetroot, has anticancer and antiproliferative activities through the induction of antioxidant defense and lipoperoxyl free radical scavenging free mechanisms [34–36]. In addition, betanin by induction of detoxifying and antioxidant enzyme expression and reduction of the xenobiotic-induced oxidative stress may play an important role in the prevention of liver, lung, and kidney injuries, and cancer [13, 37–40]. According to previous studies, it seems that red beetroot and its main constituent, betanin, and other compounds with strong antioxidant, antiproliferative and antitumor activities can be effective in the initiation and promotion stages of cancer [24, 41].

Although different in vitro and in vivo studies have shown the chemopreventive and cytotoxic activities of red beetroot in different cancers, there is limited information and studies to elucidate thorough mechanisms and cellular pathways of these actions in colorectal cancer. Therefore, the present study aims to clarify the exact mechanism of anticancer activity of red beetroot extract and betanin in colorectal cancer cell lines (Caco-2, HT-29) in comparison with normal epithelial cells (KDR/293). In this way, to explore the exact mechanism of its apoptosis-inducing effects the expression level of 6 key genes (Bcl-2, BAD, Fas-R, Caspase-3, Caspase-8, and Caspase-9) that play important roles in intrinsic and extrinsic apoptosis pathways were examined.

## Materials and methods

### Plant material

The mature beetroots were obtained from a local market in Tabriz city, Iran. Leaves were removed and soil cleaned from the roots and washed several times with sterile water, then clean roots were chopped and used for hydro-alcoholic extraction. All the processing of these samples was performed in accordance with relevant guidelines and regulations of the pharmacognosy department of Tabriz Faculty of Pharmacy, Herbarium of Faculty of Pharmacy with code: TBZFPH. Furthermore, this study was ethically approved by TBZMED (IR.TBZMED.REC.1395.703).

### BHE extraction

The process involves homogenizing the beetroots to a fine pulp and extracting the compounds using a hydro-alcoholic solvent, in this case, 70% ethanol. The extract is then centrifuged to remove any insoluble material, and the supernatant is dried at 40 °C under vacuum.

The residual aqueous was dissolved in 1000 mL of 70% methanol (300 mL water/700 mL methanol), then the methanol-sample mixture was refrigerated at − 20 °C for 24 h, and the supernatant was carefully collected from the precipitate. The methanol was removed from the supernatant by evaporation at 40 °C under vacuum, then the aqueous fraction was lyophilized (Christ, Alpha 1–2, Germany) and used as dry beetroot extract. The yield of dry extract obtained from 1 kg of beetroots is 14.5 g/1 kg, which means that the extract is approximately 1.45% of the total weight of the beetroots.

### Cell lines and culture medium

Two human colorectal cancer cell lines (Caco-2, ATCC, HTB-37 and HT-29, ATCC, HTB-38) and a human epithelial normal cell line with the same embryonic origin (KDR/293) were cultured in 25 cm^2^ culture T-flasks in Roswell Park Memorial Institute medium (RPMI 1640, Sigma, Poole, United Kingdom) and Dulbecco's Modified Eagle's Medium (DMEM, Gibco, Grand Island, NY, USA) respectively under standard conditions at 37 ^ºC^ and 5% CO2.

All media were supplemented with 10% (v/v) fetal bovine serum (HyClone, Logan, UT, USA), 1% of mixture penicillin (100 IU/ml) (Sigma, St. Louis, MO, USA), and streptomycin (100 µg/ml) (Sigma, St. Louis, MO, USA).

### MTT assay

**Fig. 2:**
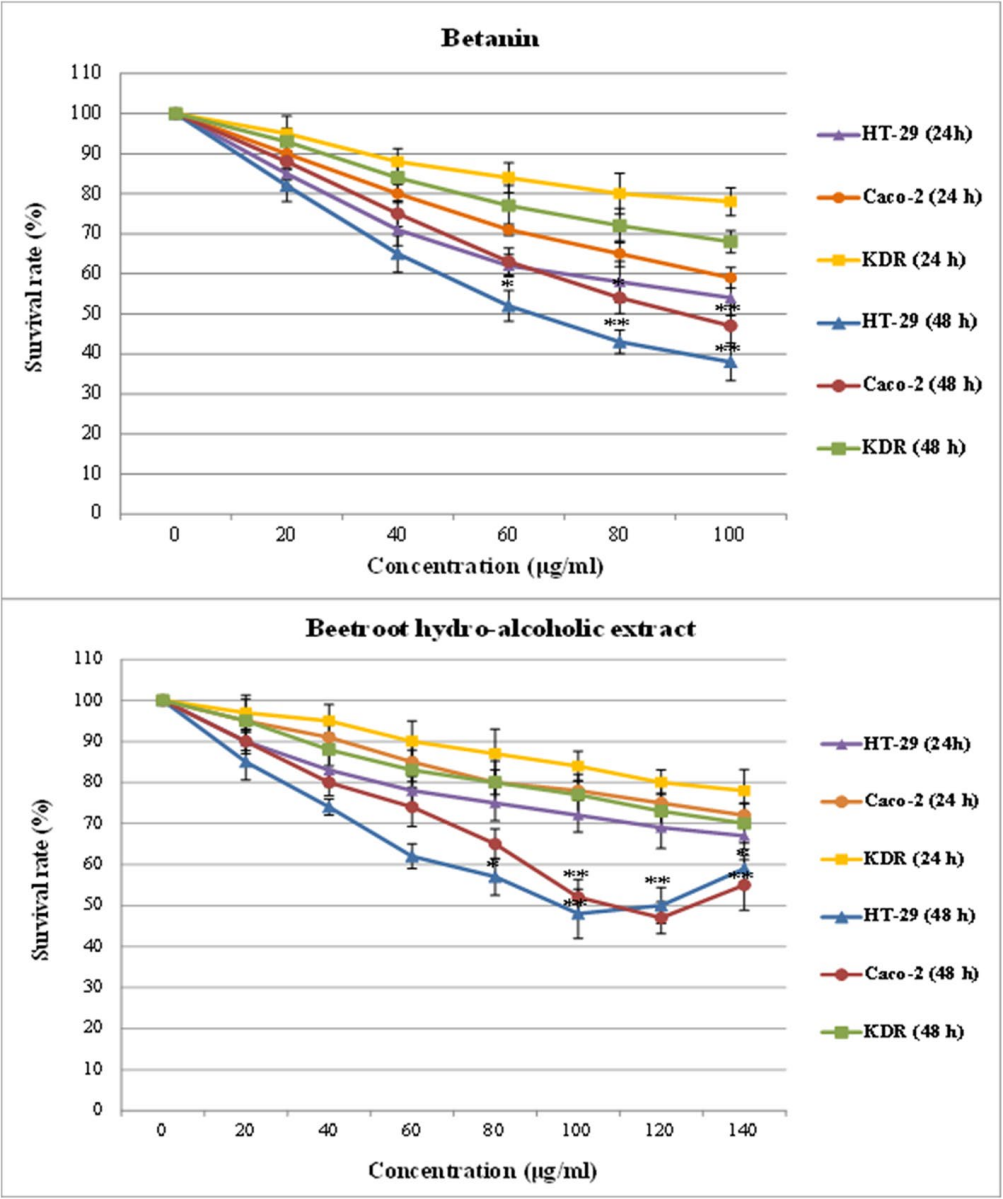
MTT assays were used to determine half maximal inhibitory concentration (IC50) in HT-29 and Caco-2 cells after treatment with different concentrations of betanin and BHE were determined about 92 μg/mL, 107 μg/mL and for betanin about 64 μg/mL, 90 μg/mL for HT-29 and Caco-2 cells at 48 h, respectively. Treatment with betanin significantly decreased survival rate in HT-29 and Caco-2 cells while KDR/293 normal cells remained intact. Data were obtained from 3 independent experiments for each test and were normalized to naive cells and presented as percent viable cells (means ± SD). Asterisks indicate a significant increase in cell death (**p ≤ 0.01, *P < 0.05)

In this study, the MTT test was performed on both untreated and treated cell lines using BHE, betanin (Sigma-Aldrich Chemie GmbH), and 5-Fluorouracil (5-FU) (5-FU) (7 μl/well) as described in our previous studies [42, 43]. The IC50 were determined in HT-29 and Caco-2 cells by prescreening MTT tests (in the range of 20 to 140 µg/ml) at 24 and 48 h (Fig. 2). The absorbance was measured using ELISA plate reader (Biotek, ELx 800, USA) at 570 nm. The growth inhibitory effects of the supernatant were calculated using the Growth Inhibition Ratio (IR%) formula, which is based on the percent of difference in absorbance between the blank control group and the experimental group.

### Morphological analysis

We applied DAPI staining method to find morphological changes regarding both treated and untreated groups with BHE, betanin, and 5-FU as exactly described in our previous studies [42, 43].

### Flow cytometer assessment

Colorectal cancer cell lines (HT-29 and Caco-2) and normal KDR/293 cells were seeded into six-well culture plates (1.2 × 10^5^ cells/well) and treated with BHE, betanin, and 5-FU. After treatment time point, the treated/untreated cells were detached by Trypsin–EDTA (Sigma-Aldrich, St Louis, MO), and the liquid phase including BHE and betanin was discarded after centrifugation at 900 rpm for 10 min at 28^ °C^. In accordance with kit instructions (eBioscience, San Diego, CA), the cell pellets were washed by phosphate buffered saline (PBS) and 1X Binding Buffer (1 mL 10X Binding Buffer + 9 mL dH2O) then centrifuged and their supernatants were thrown away. Afterward, 5 µl of FITC-conjugated Annexin V were added to 100 μl of the cell suspension and incubated for 15 min at room temperature under dark conditions. Finally, 5 µl of propidium iodide staining solution was added to the cells and flow cytometry assessment was performed on all untreated/treated cells as exactly described in our previous studies [42, 43].

### RNA isolation, complementary DNA synthesis, and real-time PCR analysis

All treated/untreated cells were washed 3 times with sterile PBS (pH 7.2). Total RNA was isolated from cells by direct lysis using RNX-plus solution according to manufacturer's instruments. The obtained total RNA pellet was dissolved in 50μL DEPC-treated water, and then quantity and quality of total RNA was assessed by UV spectrophotometry and agarose gel electrophoresis, respectively.

One microgram of isolated RNA was used for synthesis of complementary DNA (cDNA) using Prime Script RT Reagent kit according to manufacturer's recommendations for cDNA synthesis. Gene-specific primers were designed for each gene of interest, listed in the Table 1. All amplification reactions were performed in triplicate for each sample, and every experiment mixture (20 μL), containing 10 μL SYBR Green PCR master mix, 1 μL cDNA (1 μg/μL), 1 μL primer (forward and reverse), and 0.8 μL 6-carboxy-X-rhodamine (ROX as reference dye), was subjected to ABI-step I plus (Applied Biosystems, Foster City, CA, USA) instrument [44, 45]. Thermal cycling condition was as follows: 1 cycle at 95 °C for 5 min followed by 40 cycles at 95 °C for 20 s, 60 °C for 35 s, and 72 °C for 10 s. Interpretation of the results were performed using Pfaffle method and the threshold cycle (Ct) values were normalized to the expression rate of GAPDH as a housekeeping gene [46]. All of the reactions were performed in triplicate and negative controls were included in each experiment.

**Table 1:** Primers sequences for RT-PCR amplification

Gene name and symbol	Sequence (5′⟶ 3')	Amplicon size (bp)	TM	
			F	R
Bcl-2	F:5´-GGTGGGGTCATGTGTGTGG-3´ R:5´-CGGTTCAGGTACTCAGTCATCC-3´	89	60.6	60.1
BAD	F:5´-TGGACTCCTTTAAGAAGGGAC-3´ R:5´-CAAGTTCCGATCCCACCAG-3´	113	56.6	57.8
Fas R	F: 5´-AGCGCTGAAGAGCCAACATA-3´ R: 5´-TGGGTACTTAGCATGCCACT-3´	126	59.7	58.7
Caspase- 3	F:5´-TGCCTGTAACTTGAGAGTAGATGG-3´ R:5´-CTTCACTTTCTTACTTGGCGATGG-3´	172	59.8	60.1
Caspase- 8	F: 5-ACATGGACTGCTTCATCTGC-3´ R:5´-AAGGGCACTTCAAACCAGTG-3´	123	58.2	58.6
Caspase- 9	F:5´-TGCTGCGTGGTGGTCATTCTC-3´ R:5´-CCGACACAGGGCATCCATCTG-3´	94	63.2	63.1
GAPDH	F:5’-AAGCTCATTTCCTGGTATGACAACG-3’ R:5’-TCTTCCTCTTGTGCTCTTGCTGG-3’	126	61.6	62.6

### Statistical analysis

The data analysis was conducted by utilizing the statistical software package known as the Statistical Package for the Social Sciences (SPSS Inc. Chicago, IL, USA version 16.0). The normal distribution of data was evaluated using the Kolmogorov–Smirnov test. To determine the differences between all treatments and multiple mean comparisons, one-way ANOVA and Tukey's post hoc test were utilized, respectively. Statistical significance was defined as *P* ≤ 0.05, and the results were reported as means ± standard deviation (SD).

## Results

### Cell viability assay in cancerous and normal cell lines

**Fig. 3:**
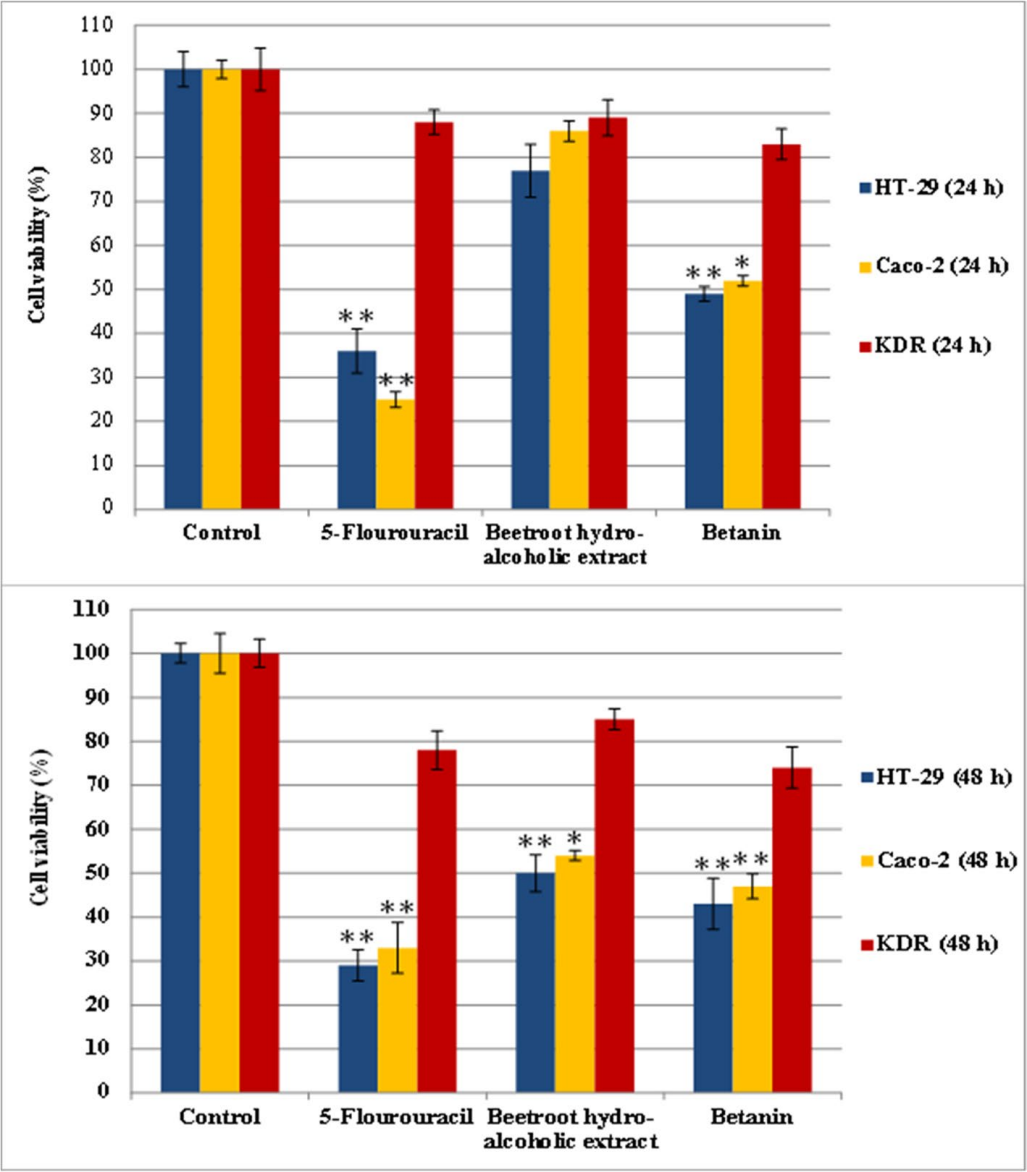
Effect of BHE and Betanin on the viability of HT-29, Caco-2 and KDR/293 cell lines. Cell viability was expressed as the percentage of optical density of the treated cells in comparison with untreated controls (100% viability). All the experiments were performed in triplicate (*n* = 3), and the data were presented as means ± SD. *P ≤ .05 and **P ≤ .01 indicate significant and highly significant vs the control group

After treatment with different doses (20 to 140 µg/ml) of BHE and betanin in two time-points (24 and 48 h), the IC50s were determined as 92 μg/mL, 107 μg/mL and 64 μg/mL, 90 μg/mL in HT-29 and Caco-2 cell lines at 48 h, respectively (Fig. 2). Also, treatment of KDR/293 normal cells (control group) with the highest determined concentration (140 μg/mL) at 24 h and 48 h time points didn't show cytotoxic effects. As shown in Figs. 2 and 3, BHE and betanin significantly inhibited the growth of HT-29 and Caco-2 cell lines in a time and dose-dependent manner (with increasing concentrations from 40 to 100 μg/mL for betanin and 60 to 100 μg/mL for BHE). Also, the lowest concentration amounts that significantly inhibited the growth rate of cancerous cells were determined as 60 μg/mL and 100 μg/mL for betanin and BHE at 48 h respectively (*p* ≤ 0.01). Moreover, treatment with a higher concentration of BHE increased the survival rates of HT-29 (at concentrations more than 100 μg/mL) and Caco-2 (at concentrations more than 120 μg/mL) cell lines at 48 h.

### Qualitative apoptosis assessment (DAPI staining)

**Fig. 4:**
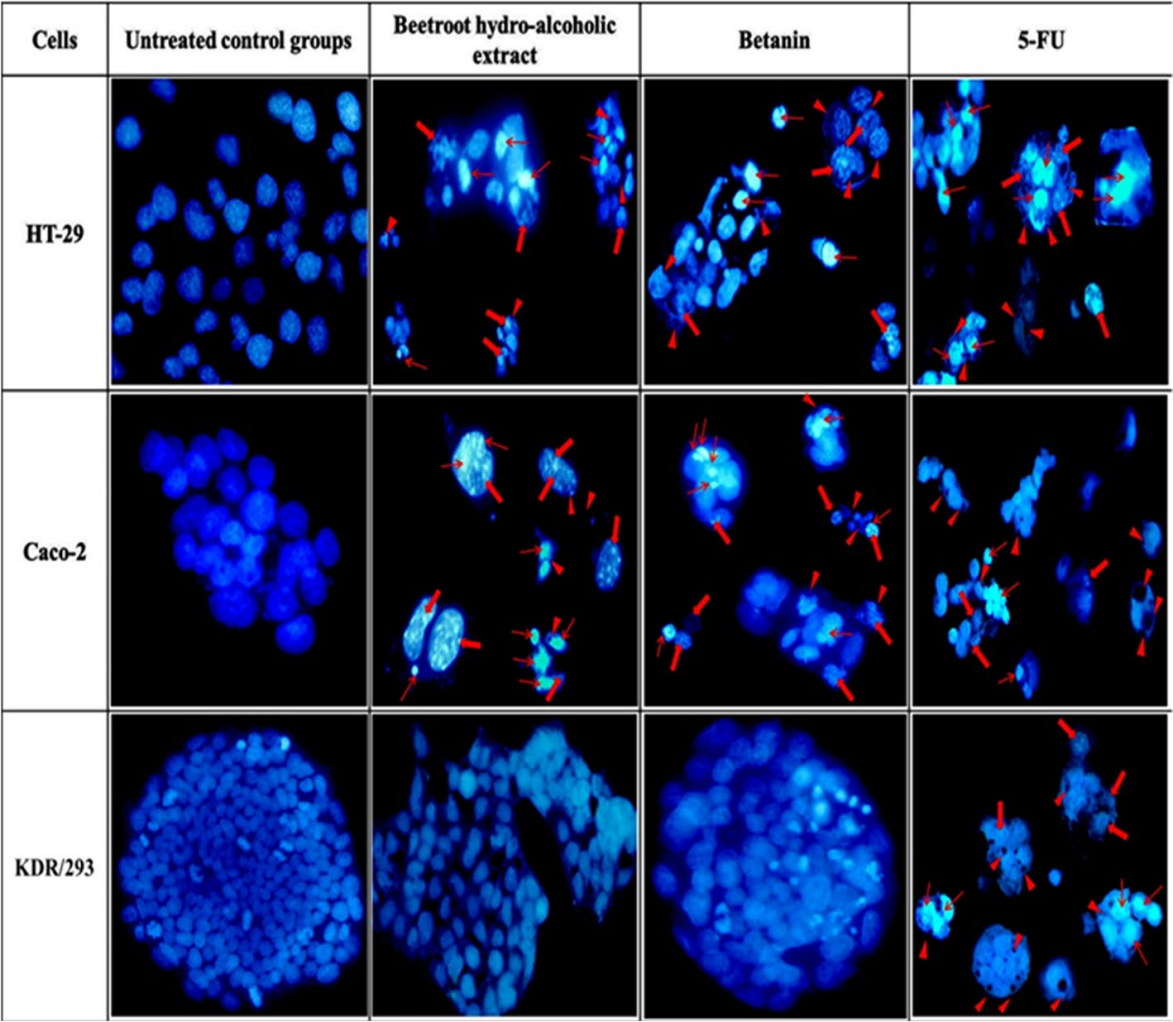
DAPI staining of treated/untreated HT-29 T, Caco-2 and KDR/293 cells. Panels represent untreated control groups, treated with BHE, treated with Betanin and treated with 5-FU (105 µl/well) as positive control groups for 48 h incubation, respectively. Arrows depict chromatin condensation (thin arrows), fragmented nuclei (thick arrows) and membrane blebbing (arrowheads)

Different apoptosis symptoms in nuclei and membrane of the cells were observed after treatment with BHE, betanin, and 5-FU as the positive control group. As shown in Fig. 4, HT-29 and Caco-2 cells that treated with BHE (92 μg/mL for HT-29 and 107 μg/mL for Caco-2), betanin (64 μg/mL for HT-29 and 90 μg/mL for Caco-2), and 5-FU (105 μL/well of 6-well plate) underwent condensed (early apoptosis) or fragmented (late apoptosis) nuclei and cell volume shrinkage, whereas whole control cells appeared as intact nuclei and membrane and were in a normal state.

### Quantitative apoptosis assay (flow cytometry)

**Fig. 5:**
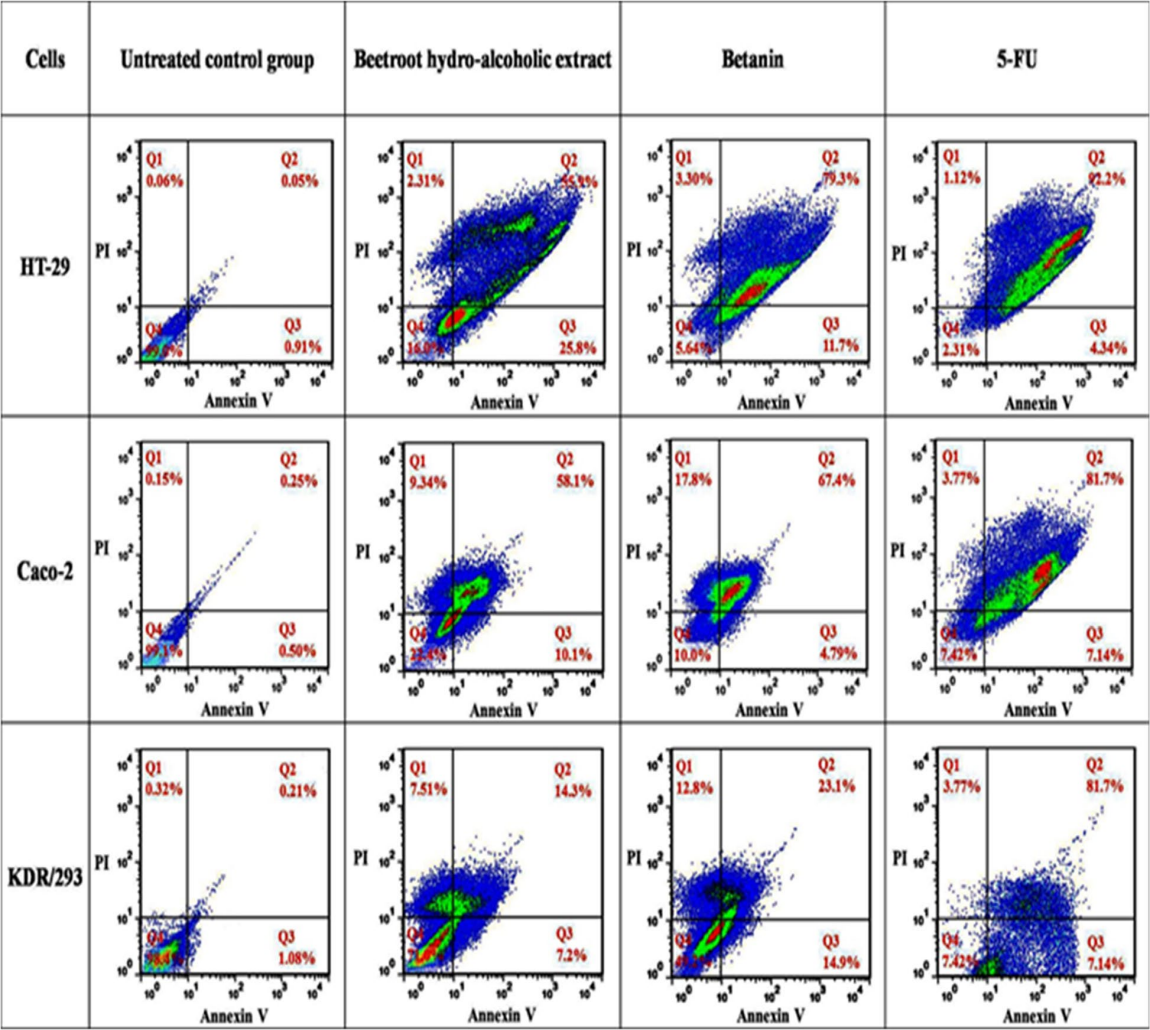
Flow cytometry analysis of treated/untreated cancerous and normal cells. Cells were treated with FITC-Annexin V in combination with PI to detect apoptosis and necrosis before being subjected for analysis by flow cytometry. Panels represent untreated control groups, treated with BHE, treated with betanin and treated with 5-FU (105 μL/well of 6-well plate) as positive control groups for 48 h incubation, respectively. Dots with Annexin V**-**/PI + (Q1), Annexin V + /PI + (Q2), Annexin V + /PI**-** (Q3), and Annexin V**-**/PI (Q4) and feature represent necrotic, late apoptotic, early apoptotic, and viable intact cells, respectively

Treatment with BHE and betanin significantly increased percentage of cells in early (Annexin V + /PI-) and late (Annexin V + /PI +) apoptosis in HT-29 and Caco-2 cancer cell lines after 48 h compared with the untreated control and normal KDR/293 cells that showed less cell death (Fig. 5). The total percentages of early and late apoptosis ratio after treatment with BHE and betanin for 48 h in the HT-29 and Caco-2 cell lines were found to be 81.7%, 91%, and 68.2%, 72.1% respectively. Also, in the positive control group (KDR/293 normal cells), the apoptosis ratio ware determined as 21.5% and 38% respectively. Based on these findings, it seems that the apoptosis-inducing effect of betanin in cancerous and normal cell lines was more than BHE. However, the apoptotic effects of betanin and BHE on HT-29 and Caco-2 cell lines were comparable with 5-FU as an approved anticancer drug and these effects in KDR/293 cells were less than 5-FU.

### Gene expression levels

**Fig. 6:**
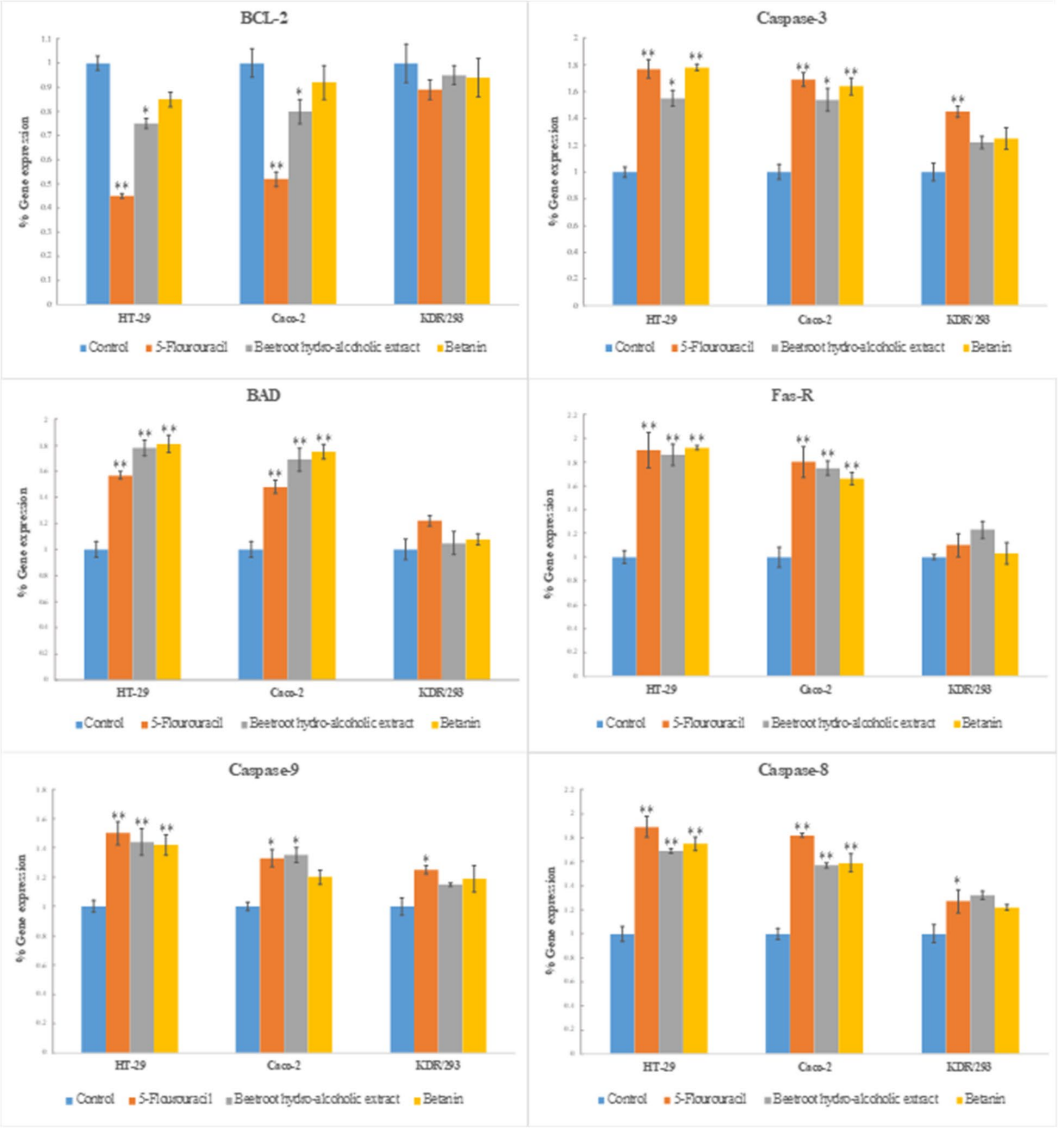
Expression level of key apoptosis pathway genes in the HT-29, Caco-2, and KDR/293 cancer cells lines after treatment with BHE, betanin, and 5-FU (105 μL/well of 6-well plate) as positive control group for 48 h incubation. Target genes were normalized to GAPDH as housekeeping control gene (^*^p ≤ 0.05, ^**^p ≤ 0.01 as compared to control)

As shown in Fig. 6, treatment of HT-29 and Caco-2 cell lines with BHE and betanin for 48 h resulted in up-regulation of proapoptotic genes such as BAD, Fas-R, Caspase-3, Caspase-8, and Caspase-9. On the other hand, the expression level of the anti-apoptotic gene Bcl-2 was decreased significantly in both cancer cell lines after treatment with BHE. Interestingly, the expression level of proapoptotic gene BAD, in the HT-29 and Caco-2 cancer cells after treatment with BHE and betanin was even higher than 5-FU as a positive control group. Moreover, the upregulation level of proapoptotic genes, Fas-R, Caspase-3, Caspase-8, and Caspase-9 were comparable with 5-FU.

Our findings showed that BHE and betanin can stimulate the intrinsic and extrinsic apoptosis pathways in HT-29 and Caco-2 cancer cell lines via downregulation of Bcl-2 and upregulation of BAD and Caspase-9 genes and also upregulation of Fas-R, Caspase-3, and Caspase-8, respectively. In addition, the expression level of apoptotic genes in KDR/293 cells did not significantly change after treatment with BHE and betanin.

## Discussion

Cancer is a major cause of death globally, and reducing chronic diseases could lead to better health and longer life [47]. Studies from Europe and the USA have shown that consuming more fruits and vegetables is associated with a lower risk of death [48, 49]. Research indicates that consuming fruits and vegetables may help prevent chronic diseases such as cancer [47, 50]. Betalains, natural antioxidants with potential health benefits, are being considered by supplement manufacturers [51, 52]. Red beetroot extract (*Beta vulgaris* L.), which is approved by the food and drug administration (FDA) as a red food color (E162), has been shown to reduce the incidence of experimental tumors in several organs and is a promising natural product for preventing and treating human cancers [24, 41]. Also, beetroot is potentially chemopreventive by delay in tumor onset, increase of tumor latency, decrease in tumor multiplicity, ROS release, and additional reduction in splenomegaly [24, 29, 53, 54].

Although there have been various studies conducted on the potential anticancer effects of beetroot and betanin, very few studies have been conducted on their molecular pathways and anticancer activities in colorectal cancer. As a result, we conducted a study to determine the anticancer properties of beetroot and betanin in colorectal cancer cell lines, as well as their associated molecular pathways. Our results indicated that both BHE and betanin can inhibit cell proliferation in colorectal cancer cells by inducing apoptosis through the modification of certain key genes, at different doses. Conversely, KDR/293 cells, which served as the normal control group, were unaffected by BHE and betanin at similar doses and time points.

The findings of our study demonstrated that at 48 h, BHE at a concentration of 92 μg/mL and 107 μg/mL inhibited cell proliferation in HT-29 and Caco-2 cell lines, respectively. Moreover, betanin induced cell apoptosis at lower concentrations than BHE in HT-29 (64 μg/mL) and Caco-2 (90 μg/mL) cell lines without significant effects on apoptosis of normal KDR/293 cells.

Several previous studies have demonstrated that BHE (*Beta vulgaris* L) possesses potent chemopreventive activity, reducing cell proliferation, angiogenesis, inflammation, and inducing apoptosis in various cancer cell lines such as human lymphoma (Raji cells) [55], human melanoma cells (B16F10) [56], human chronic myeloid leukemia cells (K562) [26], human colonic adenocarcinoma cells (HT-29), human liver hepatoma cells (Huh7) [36], Caco-2 cell line [54], and human breast cancer cell lines (MCF-7) [23, 57]. Similarly, betacyanins and isobetanin have been shown to possess anti-inflammatory, hepatoprotective, radioprotective, neuroprotective, diuretic, hypolipidemic, osteoarthritis pain-relieving, and anti-diabetic properties in different doses and time-points, with various in-vitro and in-vivo studies demonstrating their ability to reduce cancer cell proliferation with different IC50s [26, 58]. Moreover, red beetroot extract has been found to have antiproliferative effects on androgen-independent human prostate (PC-3) and breast cancer (MCF-7) cells without causing significant adverse effects on normal human skin (FC) and liver (HC) cells [23]. Betanin, which accounts for over 95% of the total betacyanins (300–600 mg/kg), is not harmful to human umbilical vein endothelial cells (HUVECs) and normal human fibroblasts cells, even at various concentrations. It also reduces the production of reactive oxygen species (ROS), decreasing the intracellular ROS level by about threefold [14, 59], and boosting the caspase-3 activity in stimulated neutrophils at a concentration range of 100–300 mM [57, 59]. Also, betanin exerts its potential anticancer and chemopreventive effects through different mechanisms such as; induction of antioxidant defense, free radical scavenging, scavenging DPPH-, galvinoxyl-, superoxide-, and hydroxyl-radicals, induction of Nrf2 transcription factor that leads to elevation of heme oxygenase-1 (HO-1) protein levels, paraoxonase-1 (PON-1) transactivation and cellular glutathione (GSH) [34, 36]. On the other hand, some studies showed that betanin, as the main beetroot pigment, increases the apoptosis in Ovarian cancer cell line (PA-1) and human glioma cells (U87MG) via the formation of intracellular ROS that leads to decreased mitochondrial membrane integrity and release of cytochrome *c* to the cytosol [60, 61]. Although betanin is known as a potent antioxidant in normal cells, it can act as a pro-oxidant compound in cancer cells and catalyze DNA damage through the release of ROS by reducing Cu (II) to Cu (I). However, the treatment of polymorphonuclear neutrophils (PMN) with similar amounts of betanin had no significant effect on DNA damage [54, 62].

In addition, betanin leads to death in cancer cells by increasing or decreasing the expression of some genes related to apoptosis. For example, treatment of MCF-7 cells with betanin-enriched red beetroot (*Beta vulgaris* L.) extract led to an increase in the expression of apoptosis-related proteins such as Bad, TRAILR4, FAS, and p53 and changes in the mitochondrial membrane potential. These modifications confirm the participation of both the intrinsic and extrinsic apoptosis pathways following red beetroot extract treatment [57]. However, beetroot extract has been shown to have lower toxicity on normal cells when compared to the well-known chemotherapeutic agent, doxorubicin (Adriamycin) [23]. Another in-vitro study by Sreekanth et al*.* demonstrated that betanin, isolated from the fruits of *Opuntia ficus-indica* reduced cell proliferation of human chronic myeloid leukemia cell line (K562) with an IC50 of 40 μM. Additionally, betanin was found to induce intrinsic apoptosis pathway mediated by the release of cytochrome *c* from mitochondria into the cytosol, poly (ADP) ribose polymerase (PARP) downregulation of Bcl-2, reduction in the membrane potentials, and qualitative chromatin condensation, cell shrinkage, and membrane blebbing [26]. Similarly, our study indicated that both BHE and betanin can inhibit cell proliferation and induce apoptosis in treated HT-29 and Caco-2 cancer cell lines.

Furthermore, some in-vivo studies reported that the drinking water of red beetroot and betanin on the female mice with lung cancer led to reduce tumor multiplicity (20%) and tumor load by inhibiting the angiogenesis and increasing the expression level of caspase-3 resulted in induction of apoptosis. As well, betanin stimulated apoptosis by induction of procaspase-3 cleavage and activating caspase-3, -7, -9, and PARP in Caco-2, and human lung cancer cell lines [22, 54]. In study by Lechner et al*.* was observed regular oral consumption of red beetroot (78 μg/mL) reduced the number of NMBA-induced esophageal papilloma tumors by 45% and cell proliferation in both precancerous esophageal lesions and in papilloma of NMBA-treated rats. Moreover, in animal model, the consumption of beetroot color led to decrease angiogenesis and inflammation, and to induce apoptosis [41].

Numerous studies have confirmed the beneficial effects of red beetroot and betanin on various cancers. However, the exact mechanisms behind these effects remain unclear. Several in-vitro and in-vivo studies suggest that the chemotherapeutic and antiproliferative activities of betacyanins and betanin may be attributed to their antioxidative properties. They may reduce the level of reactive oxygen species to a minimum level, which would not stimulate proliferation through inappropriate signal transduction at that level. This is just a hypothesis based on the available evidence [63].

Apoptosis is a highly regulated process of programmed cell death that eliminates damaged cells and is critical for maintaining the normal function of tissues [64, 65]. Even small defects in this system can lead to cancer or autoimmunity, while increased apoptosis can result in degenerative diseases [66]. The inhibition of apoptosis is thought to be a key factor in the development and progression of certain cancers [67]. Malignant cells can trigger this precise mechanism through either the caspase-mediated extrinsic or mitochondrial intrinsic pathways, which activate effector caspases that cause various cellular changes such as membrane blebbing, cell shrinkage, nuclear fragmentation, chromatin condensation, chromosomal DNA fragmentation, and mRNA degradation [64, 68]. The expression levels of pro/anti-apoptotic proteins and their corresponding genes are crucial for regulating cell survival and apoptosis, and natural anticancer compounds can modulate these levels [42, 43, 69]. In the early stages of cancer development, DNA damage in precancerous cells activates apoptosis pathways to eliminate harmful cells and prevent tumor growth. However, various carcinogenic factors can disrupt this process, leading to uncontrolled cell proliferation, cancer progression, and resistance to drug treatments [70, 71].

Therefore, the previous studies suggested that the betalains are the most principal and potent anticancer constituents of the red beetroot extract [41, 72]. In the present study, we focused on betanin as the major (up to 95%) beetroot betacyanins that is the leading candidate for anticancer activity of red beetroot extract. According to our results based on qualitative (DAPI staining) and quantitative (flow cytometry) apoptosis assays, red beet root extract and betanin, with different doses, induced apoptosis pathways in both colorectal cancer cell lines and this effects is comparable with routine anticancer drug, 5-FU.

Further studies including in-vivo models and clinical trials are needed to elucidate the exact cytotoxic and antiproliferative mechanisms of red beetroot extract and its main constituent, betanin or other effective compounds with anticancer activities in different cancers.

## Conclusions

To summarize, the study found that using red beetroot extract (BHE) and betanin inhibited the growth of colorectal cancer cells (HT-29 and Caco-2) while inducing apoptosis with minimal negative impact on normal cells. However, the precise mechanisms behind these effects are still unclear and further research is required to understand the anti-proliferative and apoptotic properties of red beetroot extract and betanin on different types of cancer cells, as well as exploring other herbal remedies.

## Data Availability

The datasets used and/or analyzed during the current study are available from the corresponding author on reasonable request.
